# Transient diabetes mellitus secondary to L-asparaginase

**DOI:** 10.1186/1687-9856-2013-S1-P22

**Published:** 2013-10-03

**Authors:** Lalaine Audrey G Matitu-Untalan

**Affiliations:** 1 Philippine General Hospital, Philippines

## 

Secondary Diabetes Mellitus (DM) has been associated in about 1-14% of patients with hematologic malignancy treated with L-asparaginase. An 11-year old Filipino female, diagnosed with Acute Lymphoblastic Leukemia, developed hyperglycemia associated with polyuria and polydipsia while on-going induction chemotherapy. There was no family history of diabetes mellitus. Chemotherapy drugs included L-Asparaginase, Prednisone, Vincristine and intrathecal methotrexate using the LIC protocol. Among these agents, Prednisone and L-Asparaginase are known to cause hyperglycemia. Given the risk, the patient was monitored by checking for glucosuria at least once a week. The L-asparaginase was started on Day 8 of chemotherapy after one week of Prednisone given at 60mg/kg/m^2^. No glucosuria was noted prior to the start, as well as during the first week of chemotherapy. On Day 16 of chemotherapy, after 4 doses of L-Asparaginase at 6000u/m^2^ 3x a week, she developed epigastric pain, vomiting, polyuria, excessive thirst, hyperglycemia and glucosuria (+4). Arterial blood gas was normal and negative for urine ketones. Serum amylase and lipase were likewise normal. The Random blood sugar was 680 mg/dL (37.4mmol/L). Upon repeat the next day, it was 619 mg/dl and HbA1c at 7.4%. Regular insulin started at a dose of 0.8unit/kg/day given every six hours which was later increased to 1unit/kg/day. The patient was maintained on pre-mix Insulin (70% isophane/ 30% regular) at 1.2 unit per/kg/day with good control of blood sugar between 100-200mg/dl. L-Asparaginase was completed for 9 doses. The patient required tapering doses of insulin therapy for one month after the last dose of L-Asparaginase. After discontinuation of insulin, blood monitoring for two more weeks showed no recurrence of hyperglycemia and the associated symptoms.

Inhibition of insulin and insulin receptor synthesis, leading to a combined insulin deficiency and resistance syndrome, is the supposed mechanism of the L-asparaginase effect. The temporal relationship of the appearance and resolution of hyperglycemia with the L-Asparaginase administration and discontinuation respectively, is the strong basis for attributing the transient DM to the said agent rather than to steroids. However, the risk and severity of DM increases when L-asparaginase and steroids are used concomitantly. Close monitoring of the blood sugar levels of such patients is emphasized.

